# The Interleukin-1 (IL-1) Superfamily Cytokines and Their Single Nucleotide Polymorphisms (SNPs)

**DOI:** 10.1155/2022/2054431

**Published:** 2022-03-26

**Authors:** Payam Behzadi, Aga Syed Sameer, Saniya Nissar, Mujeeb Zafar Banday, Márió Gajdács, Herney Andrés García-Perdomo, Kulsum Akhtar, Marina Pinheiro, Peter Magnusson, Meysam Sarshar, Cecilia Ambrosi

**Affiliations:** ^1^Department of Microbiology, College of Basic Sciences, Shahr-e-Qods Branch, Islamic Azad University, Tehran 37541-374, Iran; ^2^Molecular Disease & Diagnosis Division, Infinity Biochemistry Pvt. Ltd, Sajjad Abad, Chattabal, Srinagar, Kashmir, India; ^3^Department of Biochemistry, Government Medical College, Karan Nagar, Srinagar, Kashmir, India; ^4^Department of Oral Biology and Experimental Dental Research, Faculty of Dentistry, University of Szeged, 6720 Szeged, Hungary; ^5^Division of Urology, Department of Surgery, School of Medicine, UROGIV Research Group, Universidad del Valle, Cali, Colombia; ^6^Department of Clinical Biochemistry, Sher I Kashmir Institute of Medical Sciences, Soura, Srinagar, Kashmir, India; ^7^Departamento de Ciências Químicas, Faculdade de Farmácia, Universidade do Porto, Rua de Jorge Viterbo Ferreira, 228, 4050-313 Porto, Portugal; ^8^CHUP, Centro Hospitalar Universitário do Porto, Largo do Prof. Abel Salazar, 4099-001 Porto, Portugal; ^9^School of Medical Sciences, Örebro University, SE, 701 82 Örebro, Sweden; ^10^Cardiology Research Unit, Department of Medicine, Karolinska Institutet, 171 76 Stockholm, Sweden; ^11^Research Laboratories, Bambino Gesù Children's Hospital, IRCCS, 00146 Rome, Italy; ^12^IRCCS San Raffaele Roma, Department of Human Sciences and Promotion of the Quality of Life, San Raffaele Roma Open University, 00166 Rome, Italy

## Abstract

Interleukins (ILs)—which are important members of cytokines—consist of a vast group of molecules, including a wide range of immune mediators that contribute to the immunological responses of many cells and tissues. ILs are immune-glycoproteins, which directly contribute to the growth, activation, adhesion, differentiation, migration, proliferation, and maturation of immune cells; and subsequently, they are involved in the pro and anti-inflammatory responses of the body, by their interaction with a wide range of receptors. Due to the importance of immune system in different organisms, the genes belonging to immune elements, such as ILs, have been studied vigorously. The results of recent investigations showed that the genes pertaining to the immune system undergo progressive evolution with a constant rate. The occurrence of any mutation or polymorphism in IL genes may result in substantial changes in their biology and function and may be associated with a wide range of diseases and disorders. Among these abnormalities, single nucleotide polymorphisms (SNPs) can represent as important disruptive factors. The present review aims at concisely summarizing the current knowledge available on the occurrence, properties, role, and biological consequences of SNPs within the IL-1 family members.

## 1. Introduction

Cytokines—as nonstructural and small proteins—are categorized into a variety of categories comprising of chemokines, interferons (IFNs), interleukins (ILs), tumor necrosis factors (TNFs), and lymphokines [1–3]. Normally, cytokines are immune-polypeptides or immune-glycoproteins which act as signaling pathway inducers. These low molecular weight nonstructural proteins (5-20 kDa) are secreted by a number of subpopulations of immune cells, such as macrophages (M*Φ*s), neutrophils, natural killer (NK) cells, and T cells. These immune cells are involved in regulation, modulation, and promotion of immune responses by orchestrating cytokine-mediated activation, proliferation, development, differentiation, and growth among others [2–8], together with nonimmune, hemopoietic. and nonhemopoietic cells. Therefore, cytokines play pivotal roles as modulatory proteins in inflammatory cascade immune responses and repair systems in that they modulate the innate and adaptive immune systems. Cytokines are expressed by immune and nonimmune and hemopoietic and nonhemopoietic cancer cells [6, 7, 9–11].

NK cells belong to innate lymphoid cells (ILC) and are identified as group 1 ILCs (ILC1). Activated NK cells are able to express IFN-*γ* at high levels. The NK cells have the capability to produce two opposite receptors of inhibitory (such as CD94/NKG2A and the *Human leukocyte antigen* (HLA)-I-specific killer immunoglobulin-like receptors) and activate CD161, DNAM-1, natural cytotoxicity receptors (NCR) (comprising NKp30, NKp44, and NKp46), and NKG2D at the same time. Induction of these receptors determines the effector functions and the activities of NK cells [8, 12–16]. The effector cells of ILCs, e.g., NK cells directly contribute to defense system of human host. They are actively recruited in the processes of lymphoid organogenesis and the remodeling of secondary lymphoid organs (SLOs) by the human host immune system in opposition to tumors and pathogenic microorganisms, after birth. As the ILCs are situated upon the mucosal surfaces, e.g., the skin, these strategic cells are known as the first group of immune cells which are contributed to immune responses against pathogens [14, 15, 17–22].

According to the ILCs' transcription factors and cytokine profile, they are categorized into two major groups of helper ILCs and cytotoxic ILCs. In this regard, the NK cells belong to cytotoxic ILCs. The NK cells are able to eliminate the tumor cells and infected cells by the viral infectious agents. Furthermore, they can express and secrete a versatile of chemokines and proinflammatory cytokines [14, 15, 23–26]. NK cells, as a portion of ILC1 subset, act as memory cells. This feature belongs to adaptive immune cells. Simultaneously, the NK cells express IFN-*γ* and the ILs of 12, 15, and 18. The IL-18 is known as a member of the IL-1 superfamily [8, 27].

The majority of cytokines are functional by an autocrine, endocrine, and paracrine fashion [6, 28]. In accordance with recent studies [29–33], the mononuclear cells in human peripheral blood highly express a wide range of immunoproteins and immunomodulators including adhesins, transcriptomic molecules, versatile soluble receptors, cytokine growth factors, and chemokine and activated endothelial molecules in patients with autoimmune diseases, e.g., systemic lupus erythematous (SLE). Interestingly, the aforementioned immunomediators like monocyte chemoattractant protein-1 (MCP-1) can be recognized as influential predictive biomarkers for detection of the disease in the early stages and effective biomarkers for monitoring the process of disease treatment [33]. In comparison with serum immunoproteins (e.g., serum MCP-1 (sMCP-1)), the urinary immunomediators (e.g., urine MCP-1 (uMCP-1)) are identified as dominant biomarkers of lupus nephritis (LN) in patients with SLE. The reason of this feature is not completely clear; however, it seems that the urinary immunomediators such as uMCP-1 are the outcome of immunoinflammatory responses and probable injuries that occur in the patients' kidneys [32, 34–40].

In the mid-1940s (in 1944), cytokines were depicted as soluble factors (pyrexins/pus) which might be present together with fever, local pain, and swelling, and the term of “cytokine” was coined in a published commentary in 1974 by Stanley Cohen et al. [4, 6, 10, 41, 42]. Earlier, cytokines were named as lymphokines in an effort to differ them from monokines; however, the term cytokine was replaced with *interleukins* (ILs) [4]. This term was suggested in 1979 during the 2^nd^ International Workshop of Lymphokines, which was held in Switzerland. The term interleukin was accepted by the cytokinologists, and this new term was officially published by the Journal of Cellular Immunology in the format of a letter to the editor [2, 12, 18].

Indeed, ILs include a vast group of molecules, including a wide range of immune mediators that contribute to several immune-responses in cells and tissues.

ILs are identified as second messenger immune-glycoprotein biomolecules, encompassing a portion of mammals' cytokines [2, 43]. Earlier, it was presumed that ILs were cytokines which were produced and secreted by leukocytes only; however, this thought has changed by the progression of our knowledge in this regard. ILs are immune-glycoproteins, which directly contribute to growth, activation, adhesion, differentiation, migration, proliferation, and maturation of immune cells. To this aim, ILs are involved in pro and anti-inflammatory responses by their interaction with a wide range of receptors, e.g., Toll-like receptors (TLRs) which are involved in damage/danger-associated molecular patterns (DAMPs), microbial/microbe-associated molecular patterns (MAMPs), pathogen-associated molecular patterns (PAMPs), and xenobiotic-associated molecular patterns (XAMPs). The cytosolic section of TLRs, responsible for intracellular signaling pathways, is composed of a Toll/IL-1 receptor (Toll/IL-1R) (TIR) domain. This domain is common and identical among TLRs and engagement of ILs binds them together as a unified structure [4, 44–48]. On the other hand, ILs and TLRs are involved in homeostasis, cancers, and autoimmune and infectious diseases [2, 6, 10, 47, 48]. Because of the importance of IL biomolecules and their role in different parts of the human body, the occurrence of any mutation or polymorphism in their genes may results in a wide range of diseases and disorders. Among these abnormalities, single nucleotide polymorphisms (SNPs) can represent as important disruptive factors. Due to this knowledge, the authors of this chapter deeply focused on the properties and the role of the occurrence of SNPs within the IL-1 family members.

### 1.1. The Phylogenetic Origin and Evolution of ILs

As mentioned above, ILs are produced and secreted by a number of immune and nonimmune cells including M*Φ*s, monocytes, T (CD4+), and endothelial cells [2, 3].

According to classical classification, the immune system is categorized into two groups of adaptive (acquired) and innate sections. The innate immune system is activated by the foreign molecules at first, and then in consequence of unsuccessful defensive activity by the innate immune system, the adaptive system initiates to be activated. The adaptive immune system consists of cellular and humoral structures. The cellular adaptive immune system involves T lymphocytes including CD4+ T helper 1 (T_h1_), T_h2_, and T_h17_ which express a wide range of cytokines to remove microbial pathogens; while the humoral adaptive immune system is comprised of plasma cells and B lymphocytes which eliminate the extracellular pathogenic microorganisms through expression of antibodies [49, 50].

In recent decade, a new group of cells including ILCs is detected which is determined based on phenotypic characteristics and cytokine profile associated with subsets of CD4+ T lymphocytes. In this regard, the ILC family involves three members which are detected of three subsets of ILC1, ILC2, and ILC3. Moreover, recently a new subpopulation of ILCs with regulatory functions are identified which is named as regulatory ILCs (ILCregs). ILCregs resemble the regulatory subset of T lymphocytes (Tregs) and it has been shown that each subset of ILCs has its own function, transcription factors, and cytokine production [49, 51–53]. As previous reports show, the ILCs are sensitive to stress and microbial compartments. Therefore, the ICLs react to signals caused by stress in adjacent cells within a tissue microenvironment and microbial agents (e.g., intra and extracellular microorganisms and parasites) through expression of cytokines. In addition, the ILCs are involved in tissue homeostasis and modeling (as active regulators), human body's temperature, metabolism (lipid), inflammation responses, and pathogenesis (including autoimmune diseases, allergy, asthma, and cancers) [54–59].

In accordance with previous studies on lower invertebrates, the development of innate immune system is older than the adaptive immune system. Their immunity mechanisms work through the expression of antimicrobial peptides, phagocytes, pattern recognition receptors (such as TLRs), and reactive oxygen species (ROS) [60, 61]. In this regard, the ancient jawless fish of lamprey and hagfish were identified as the first vertebrates armed with an adaptive immune system. Based on genomic and proteomic studies, lamprey and hagfish possess analogous adaptive immune cells, similar to B and T cells from vertebrates. These are known as lymphocyte-like cells (LLCs) that express variable lymphocyte receptors (VLRs). Although VLRA and VLRB receptors are shared in both of jawless fish of lamprey and hagfish, the LLCs in lamprey are able to express a third receptor, VLRC. Indeed, there is close resemblance between VLRA, VLRB and VLRC and B- and T cells receptors in higher vertebrates [61–64].

The phylogenetic origin of IL families lays from more than one ancestor. As shown by phylogenetic analyses, IL families are composed of heterogenous category of proteins. The IL families are originated from more than one ancestor, for instance, as conserved sequences indicate, IL-28 has a close homology to the ancestral sequence of basal cell adhesion molecule (BCAM). On the other hand, the IL-4 molecule was first emerged in fungi, while IL-6 underwent a rapid evolutionary process with new functional characteristics. However, the ancestral sequences of IL-4 and IL-6 have a close homology to the sequences of transforming growth factor-*ß*1 (TGF-*ß*1) [61].

As Kubick et al. showed in their investigation, a clear heterogeneity was detected among IL family members. The lowest degree of heterogeneity belongs to the IL-1 and IL-6 family members. They display a high homology rate (99.92%) with TGF-*ß*1 [61]. Conversely, the highest score of heterogeneity was seen among IL-17 family members. Members of this family originated from five putative ancestors, comprising TGF-*ß*1, Spaetzle/Spätzle (SPZ), zinc finger CCCH*-*type containing-3 (*ZC3H3*), prothoracicotropic hormone (PTTH), and NOGGIN protein, with a homology score of 99%, 98%, 97.1%, 96.5%, and 95%, respectively. Instead, IL-2, IL-4, IL-10, and IL-28 family members have different putative ancestors [61].

Although results from previous studies showed that T and B cell receptors are absent in jawless vertebrates, VLRs are functional adaptive immune elements in these low vertebrates. The *VLR* genes are encoded in a genomic environment involving leucine-rich-repeat (LRR) cassettes. The antigenic receptors of VLRA, B, and C in lamprey are similar to those receptors expressed by the *αβ*T-, B, and *γδ*T cells in adaptive immune system of the higher vertebrates, respectively [65]. On the other hand, IL receptors (ILRs) have no single ancestor. In other words, ILRs resembling ILs are heterogenous proteins. Although, ILs were emerged in fungi, they continued their evolutionary process, thereby emerging in higher vertebrates, e.g., mammals. Despite the presence of a high rate of heterogenicity in both IL proteins and *IL* genes, ILs are unified as pivotal immune-modulators in regulating T cells' activities and functions [61].

## 2. Genomics of ILs

Because of the importance of immune system in different organisms, the genes belonging to immune elements, such as ILs, have been studied rigorously. The results of recent investigations showed that the genes pertaining to the immune system undergo the evolutionary process with a constant rate. This feature is driven through helpful and effective mutations within these genes, which may lead to a coevolutionary adaptation in host-pathogen interactions. In this regard, the results of a survey showed that out of 25 genes with highest evolutionary divergence in humans, seven genes belong to cytokines (e.g., ILs) [43, 66]. Interestingly, despite the occurrence of evolutionary divergence among cytokine genes (such as *IL* genes), some structural resemblance can still be seen. The reason of this resemblance is the rapidity of the evolutionary mechanisms that occurred via several duplications. Moreover, the occurrence of whole genome duplications promoted harmful and destructive mutations. During these processes, some genes disappeared, while others changed their functions acquiring new activities. Therefore, gene neofunctionalization, gene subfunctionalization, and changes in its ancestral activities may lead to genome diversification [43, 66].

**Figure 1:**
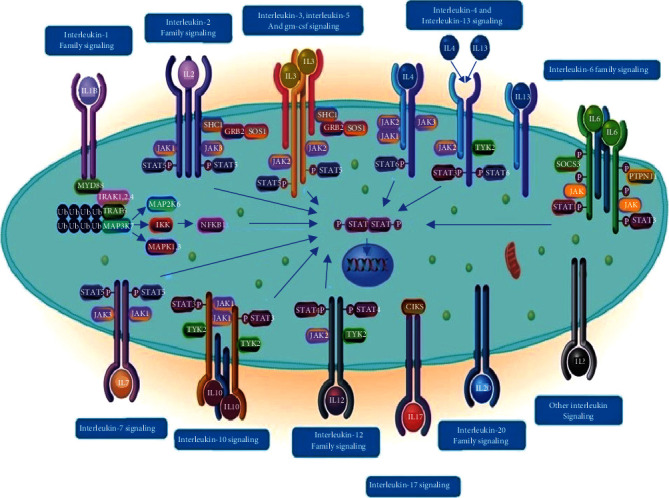
A schematic figure of low molecular weight of interleukins bound to their specific receptors located upon the cell membrane which induce different signaling pathways (https://reactome.org/PathwayBrowser/#/R-HSA-449147) [69, 70]. Janus kinase 1 (JAK1); *Tumor necrosis factor (TNF);* Signal transducer and activator of transcription (STAT); Phosphorylated STAT (pSTAT); TNF receptor-associated factor 6 (TRAF6); Mitogen-activated protein kinase 14 (MAPK14); c-Jun N-terminal kinases (JNKs); Extracellular signal-regulated kinases (ERKs); Mitogen-activated protein kinases (MAPKs); Phosphatidylinositol 3-kinases (PI3Ks); Granulocyte-macrophage colony-stimulating factor (GM-CSF); Pulmonary alveolar proteinosis (PAP); Suppressor of cytokine signals (SOCS); Protein Tyrosine Phosphatase Nonreceptor Type 11(*PTPN11*); Cytosolic adaptor molecule ACT1 (also known as CIKS); Tyrosine Kinase 2 (*TYK2*); IL1R-associated kinase (IRAK); SHC Adaptor Protein 1(SHC1); Growth factor receptor-bound protein 2 (GRB2); IkappaB kinase (*IKK*); Myeloid differentiation primary response 88 (*MYD88*).

As previous reports showed, the arrangement of the *IL* genes depicts their genomic characteristics. For instance, the presence of *IL-1* family genes located within a cluster made up of 430 kilobases (kb) on human genomic chromosome 2 (excluding *IL-18* and *IL-33* genes) underlines that they belong to a similar ancestral gene (Figure 1). However, the ancestral gene has undergone several duplications over time [43]. Moreover, the occurrence of gene duplications can be identified in both *IL-2* and *IL-21* genes. These genes are located on human genomic chromosome 4 with a distance of >244 kb [43].

### 2.1. ILs' General Characteristics and Classification

Up to date, there is no appropriate universal system associated with cytokines including ILs' categorization. ILs as a part of cytokines act as influential mediators through binding to their specific receptors. The main criteria that determine interleukins' family members include functional characteristics, biological efficacies, recruited mechanisms of action, sequence homology, and receptor chain similarities. Moreover, the structural properties of cytokines (e.g., ILs) are pivotal criteria to classify them into different groups of cytokines [11, 67, 68]. Cytokines are divided into two categories: type/class I and type/class II cytokines.

Type I ILs possess a four-helix bundle, joined together by one short and two long loops. These four antiparallel compact blocks of *α*-helices are oriented with up-up/down-down directions [2, 42, 43]. Additionally, the length of the four-*α*-helix bundle, short or long, divides the type I cytokines (ILs) into two groups: those containing more than 165 amino acids (AAs) and those with less than 165 AAs. In this regard, the short-chain type I cytokines include IL-2, IL-3, IL-4, IL-5, IL-7, IL-9, IL-13, IL-15, IL-21, colony-stimulating factor-2 (CSF-2), and thymic stromal lymphopoietin (TSLP), while the long-chain type I cytokines include IL-6, IL-11, IL-12A, IL-23A, IL-27A, IL-31, CSF-3, oncostatin M (OSM), leukemia inhibitory factor (LIF), cardiotrophin-1 (CTF-1), ciliary neurotrophic factor (CNTF), and cardiotrophin-like cytokine factor-1 (CLCF-1) [2, 43].

Type II ILs encompass a six- to seven-helix bundle, bearing six to seven compact blocks of *α*-helices with an antiparallel spatial configuration. Type II ILs include IL-10, IL-19, IL-20, IL-22, IL-24, IL-26, IL-28A, IL-28B, and IL-29 [2, 43].

The ILs of different structures identify their specific receptors and bind to them to induce the cascade of intracellular signaling pathway (Figure 1). Moreover, their activities are time-dependent, and their functions may be limited through the neutralizing antibodies and soluble receptors [10, 69, 70]. The ILs' productions are self-limited and transient. They are able to switch different signaling pathways in different immune cells (including innate and adaptive immune cells), such as phagocytes, B, and T cells, simultaneously. ILs can tune their own expression and production, as well as the activities. The ILs are also induced via cellular stimulations (e.g., B cells). They act as operators which can affect different cells enabling them to express ILs either in their neighborhood or far from them [44].

## 3. Methods and Techniques for Determining Cytokines

With the advent of advancements in techniques and tools in different disciplines such as molecular immunology, we are able to determine cytokines within different clinical samples from fluids to tissues. The goal of examination and the measuring items determines the technical procedures and the related methods [8, 11, 71]. In this regard, the analytical methodologies for cytokine determination are categorized into three groups of cytokine production analyses (involving mass cytometry, mass spectrometry, enzyme-linked immunosorbent assay (ELISA), immunoassays (multiplex), microsphere-based method, Western blotting, and enzyme-linked immunospot (ELISPOT)), intracellular cytokine analyses (comprising flow and mass cytometry, cytometric assay, mass spectrometry, Western blotting, microsphere-based method, and microsphere-based flow (multiplex)), and cytokine gene analyses (consisting polymerase chain reaction (PCR), quantitative PCR (qPCR), PCR-RFLP, microarray, and next generation sequencing (NGS)) [11, 72–77].

ELISA is known as the gold standard measurement technique for cytokine biomarkers in serum of patients with cancer and many other diseases [78–80]. Remarkably, the level of cytokines varies in different types of clinical samples (including cerebrospinal fluid, serum, cystic fluid, and plasma) and sample sites. The items of tumor grade, histological type, clinical status, etc. are considered in serum cytokine measurement in in vivo condition. The level of cytokine in serum can be recognized as predictive biomarkers. In addition, some members of cytokines, e.g., IL-1*ß*, have direct relationship with death and mortality among patients with sepsis and cancers. Hence, the IL-1*ß* and some other cytokines are identified as influential predictive biomarkers for death and mortality in patients with sepsis [77, 81–88].

Moreover, the cytokine productions can be measured in in vitro condition in cell cultures. In this regard, a versatile of techniques comprising ELISA, ELISPOT, Western blotting, etc. can be employed to assess the cytokine productions in cell culture supernatant. The monitoring process of cytokine production can be performed over time in different stages of experimental procedures [11, 89–91].

In comparison with Western blotting, ELISA is more sensitive; however, the technique of Western blotting is known as a common and appropriate detective biochemical technique for different types of proteins, e.g., cytokines (such as ILs). The Western blotting is influential quasiquantitative diagnostic tool for assessing degradation of cytokines and their precursors. Moreover, the ELISPOT technique which is categorized as high-sensitive immune assay resembles the chemiluminescent Western blotting technique. ELISPOT assay is appropriate for assessing the number of cells capable for cytokine secretion [92–95].

Flow cytometry together with mass cytometry or mass spectrometry is used as the updated tool in in vitro condition such as cell culture [96, 97]. Furthermore, the flow cytometry can be recruited as an influential assessing method for measuring the expression of intracellular cytokines within different cells to detect the related cells capable to produce cytokines. The use of Brefeldin A in flow cytometry technology promotes the sensitivity of this technique. The multicolor flow cytometry, in which several fluorochromes are employed, can be used to detect a wide range of cytokines in the same or different cell subgroups. The combined technique of flow cytometry microspheres is a useful quantitative fluorescent assay which is capable to detect several types of cytokines in low level of secretion at the same time. This technique is employed for identification of SNPs etc [89, 95, 98–104].

In addition to the aforementioned techniques for assessing the cytokines, other technologies with proteomic basics such as protein macrocalculation, mass spectrometry, and mass cytometry. The simultaneous application of heavy isotopes, e.g., ^15^N, ^2^H, ^18^O, and ^13^C used for labelling in mass spectrometry increases the capacity of its quantitative characteristics. These tools need several procedures including protein extraction, enzymatic proteolysis, high performance liquid chromatography (HPLC), ionization, and analytical methodologies to handle. Due to this fact, it is recommended to employ sharp and short methodologies such as label-free techniques. The label-free technologies and isobaric tags for relative and absolute quantification (iTRAQ) are determined as effective tools in quantitative proteomics. However, the iTRAQ techniques need several stages to be handled. In recent years, the iTRAQ technology is performed across a wide range of mass spectrometry platforms [96, 102, 103, 105–115].

The shotgun proteomics analytical methodologies involving Data-dependent acquisition (DDA) and Data-independent acquisition (DIA) techniques are not routine measuring techniques of cytokines. These techniques have their own limitations through detection of ionized peptides as the only target peptide molecules. As the cytokines including ILs are known as low molecular weight proteins, they are not detectable via DDA and DIA techniques. However, these techniques are able to detect the low amount of cytokine molecules in body fluids. Besides, the final results should be interpreted by software tools [107–110, 114, 115].

By the successful progressions in the fields of epigenomics and genomics, the molecular and genomic approaches support our knowledge regarding the processes of gene regulation and molecular signaling pathways in immune-oncology. Due to this fact, the genomic analyses are based on in silico approaches, software tools in dry labs [75, 115–119]. The applications of a wide range of molecular approaches from conventional PCR, real-time PCR, reverse transcriptase PCR to NGS, and high-throughput sequencing give us an effective opportunity to detect and identify different types of cytokines in different types of samples. Furthermore, a versatile of mutations including SNPs can be recognized through the use of these molecular availabilities. The molecular technologies enable us to identify a huge number of target molecules including DNAs, RNAs, and cytokines. to diagnose a wide range of cancers, autoimmune and infectious diseases. Bioinformatics, computational biology, and different databases support these molecular approaches to have accurate interpretations from the obtained results [7, 74–76, 116–125].

### 3.1. IL-1 Family Members

The large family of IL-1 (IL-1F) with 11 members is named after its famous member IL-1, known as the fever-inducer cytokine. Indeed, the IL-1 protein is known as a soluble endogenous pyrogen or human leukocytic pyrogen. Moreover, the members of IL-1 family are identified through their specific structures bearing a fold rich in *ß*-strand [2, 9, 66, 68, 126]. IL-1 is the first IL which was discovered in 1979 [127–129]. However, the identification of IL-1 family goes back to 1940s and 1950s when Menkin, Beeson, and Atkins identified these proteins as the endogenous pyrogenic biomolecules [130–132]. Louise Barbier et al. believed that the detection of IL-1 as well as IL-1*α* and IL-1*ß* members led to “the birth of cytokine biology.” [133]

Indeed, identification of IL-1 subsequently led to detection of its receptor (IL-1R) and the TIR domain, which is shared by both IL-1R and TLRs. The IL-1R1 is known as the main receptor for IL-1*α*, IL-1*ß*, and IL-1Ra. There is a second receptor for IL-1, the “IL-1R2,” which indeed is a decoy receptor. The IL-1R2 is a soluble protein, which encompasses only the extracellular domain without an intracellular domain. This property of IL-1R2 results in signal inhibition. Decoy receptors are conserved evolutionary structures which restrict the chemokines, cytokines, and growth factors activities [45–48, 68, 128, 129, 134].

The IL-1 family members consist of seven agonists (IL-1*α* (IL-1F1), IL-1*ß* (IL-1F2), IL-18 (IL-1F4), IL-33 (IL-1F11), IL-36*α* (IL-1F6), IL-36*ß* (IL-1F8), and IL-36*γ* (IL-1F9)), three antagonists (IL-Ra (IL-1F3), IL-36Ra (IL-1F5), and IL-38 (IL-1F10)) and an anti-inflammatory cytokine (IL-37 (IL-1F7)) in which the IL-1 has the axial role in innate immune system and inflammation modulation [2, 128, 129, 135, 136].

Moreover, the IL-1 superfamily members based on the size of N-terminal domain (NTD) of propieces are categorized into three branches or subfamilies of IL-1 subfamily (composed of IL-1*α*, IL-1*ß*, IL-33, and IL-Ra), IL-18 subfamily (including IL-18 and IL-37), and IL-36 subfamily (comprising IL-36*α*, IL-36*ß*, IL-36*γ*, IL-36Ra, and IL-38) [133, 137, 138].

Therefore, the IL-1 family members, excluding IL-1Ra, possess no signal peptide and some of them are not secreted as bioactive molecules. In other words, some IL-1 family members are expressed as inactive precursors, which should be activated by enzymatic proteolysis. The IL-1 family members are naturally spread within the cell cytoplasm entirely as active and inactive precursors or active biomolecules. Each precursor is identified through its conserved consensus sequence of A-X-Asp, where the A depicts an aliphatic AA, the X is any AA, and Asp is the abbreviation for aspartic acid [134, 139]. The caspase-1 cleavage site is situated some AAs before this motif. For instance, the consensus motif A-X-Asp in the IL-1*ß* precursor, Leu-Arg-Asp, is located nine AAs before the caspase-1 cleavage site [134, 139, 140].

The IL-1 family members form a complex network with a high plasticity that orchestrate and regulate the immune responses of the innate and adaptive immune systems. Therefore, the high diversity of IL-1 family members makes them capable to contribute to different immune responses within the human body, from homeostasis to cancer, autoimmune, infectious, and inflammatory diseases, dysmetabolism, and others [126, 128, 129, 133, 138, 141–143].

On one hand, the IL-1 superfamily members are responsible for strong inflammatory responses with potential damaging effects, while, on the other hand, these members contribute to adaptive immune system by enhancing effective immune responses against antigens and promoting nonspecific resistances against infectious diseases [134]. Accordingly, the activities of IL-superfamily members are associated with the presence of TIR domain. Indeed, a close homology between the TIR domain in IL-1R1 and the TIR domain in TLRs has been identified. Moreover, both the IL-1 family members and TLR immune-glycoproteins induce the activation of lymphocytes, which play pivotal role in the adaptive immune system [2, 47, 48, 134]. Additionally, the effects of IL-1 family members are mediated through the related heterodimers of receptors comprising IL-R1, IL-R2, IL-18R*α*, IL-18R*ß*, IL-36R, IL-1R accessory protein (IL-1RAcP), single immunoglobulin (Ig) IL-1R-related molecule (SIGIRR/IL-1R8), three Ig domain-containing IL-1R-related-1 (TIGIRR-1/IL-1RAPL1) and TIGIRR-2 [126, 133, 134, 138, 144].

These heterodimeric receptors are composed of a fixed portion (primary receptor), including specific ligand binding chain involving IL-1R1, IL-18R*α*, suppression of tumorigenicity 2 (ST2) or IL-33R and IL-36R, and a ligand-dependent variable portion (secondary receptor) of IL-1RAcP requiring IL-R1, IL-33R, and IL-36R or the IL-18R*ß* chain regarding IL-18R [126, 133, 134, 138]. The *IL-1RN* gene contributes to the expression of IL-1Ra and modulation of the signal peptide and supports the secretion of IL-1Ra through the endoplasmic reticulum (ER) and Golgi apparatus [138, 145, 146].

Indeed, the related receptors are identified as immuno-glycoproteins which belong to integral plasma membrane molecules encompassing three domains of extracellular, transmembrane, and intracellular compartments. The activated receptors of interleukins (through the processes of oligomerization or dimerization) regulate a significant portion of immune responses within the cells [11, 92, 147–149]. However, the activities of cytokines can be antagonized through the contribution of soluble receptors to make changes in cytokines' response [11].

By the involvement of ligand-fixed portion (primary) receptor complex, the second receptor subunit or the ligand-dependent variable portion is recruited to complete the holocomplex. The formation of ligand-receptor holocomplex activates the cascades of intracellular signaling pathways through the induction of proinflammatory signaling. Due to this fact, this process continues via recruiting the adaptor proteins, e.g., myeloid differentiation primary response protein 88 (MyD88), which triggers the downstream protein kinases, comprising IL-1R-associated kinases (IRAKs) and tumor necrosis factor receptor-associated factor-6 (TRAF-6). Thereby, the key transcription factors such as mitogen-activated protein kinases (MAPKs), e.g., p38 and c-Jun N-terminal kinase (JNK), extracellular signal-regulated kinases (ERKs), interferon-regulatory factors, and the nuclear factor (NF)-*κ*B get activated [47, 48, 128, 133]. Activation of mentioned signaling pathways leads to innate and acquired immune-inflammatory responses, which cover the specific immune responses at tissues and/or systemic levels [6, 47, 48, 126, 129, 138, 146].

As the results of previous studies show, the cytokine receptors interact with the Janus Kinases (JAKs) family and signal transducer and activator of transcription (STAT), and therefore, the majority of cytokines recruit the JAK/STAT signaling pathway to move their own signaling molecules (Figure 1) [7, 11, 150–152]. Interestingly, the ligands of IL-1*α* and IL-1*ß* in combination with their specific heterodimeric receptors (IL-1R1-IL-1RAcP) regulate the related signal within its own signaling pathway; while the combination of IL-1*α*/IL-1*ß* with heterodimeric receptor of IL-1R2- IL-1RAcP may lead to inhibitory signals both in extra and intracellularly conditions [126, 133]. Indeed, IL-1R2 has no intracellular TIR domain; therefore, it just takes the energies from other molecules and no signal is transmitted. In other words, the IL-1R2 is known as molecular sink regarding IL-1 family members [126, 133, 145].

The IL-1R8 (SIGIRR), IL-1R9 (TIGIRR-1/IL-1RAPL1), and IL-1R10 (TIGIRR-2/IL-1RAPL2) are known as negative regulatory receptors, which contribute to limit the proinflammatory signaling pathways. The SIGIRR is the only member of IL-1 family receptors, which possesses a single extracellular Ig domain and specific AA substitutions within the inner portion of its TIR domain. The IL-1R8 transfers inhibitory signals, which are devoted to natural killer (NK) cells maturation and regulatory activities. The interference of IL-1R8 activity with the TIR domain oligomerization activity of IL-1 family receptors, in the presence of a ligand agonist, results in blocking the involvement of the MyD88 adaptor [126, 128, 133, 136, 153–155].

The complex of SIGIRR and IL-18R*α*, IL-37R, in combination with its ligand—the IL-37—is capable of decreasing the expression of proinflammatory cytokines, such as interferon-*γ* (IFN-*γ*), tumor necrosis factor-*α* (TNF-*α*), IL-1*α*, IL-1ß, IL-1Ra, IL-8, IL-17, and IL-23. In other words, the IL-1R8 and IL-18R*α* complex acts as an immunosuppressive IL possessing an anti-inflammatory function [126, 133, 156, 157].

Up to now, eight IFN species are determined in human being from which, IFNs-*α*, *ß*, *ε*, *κ*, *υ*, and *ω* are categorized as type I IFN, IFN-*γ* is classified as type II IFN and IFN-*λ* is grouped as type III IFN molecules [11, 158–160].

In accordance with results from previous studies, there is a close homology between IL-1R9, IL-1R10, and IL-18-binding protein (IL-18BP), which may result in performing the same negative regulatory functions [126, 136]. Moreover, soluble forms of IL-1 receptors, e.g., IL-1R1 (sIL-1R1) and sIL-1R2, neutralize their specific ligands, including IL-1*α* and IL-1*ß*, to inhibit their positive role in proinflammatory signaling pathway. The same feature happens when IL-33 and IL-18 bind to soluble forms of cognate receptors, sST2 and IL-18BP, respectively [126, 133, 134, 145].

### 3.2. IL-1F Members and Trained Immunity

When the host's innate immune system faces pathogenic microorganisms and microbial products unconsciously (by accident) or consciously (by vaccination), it results in the appearance of de facto memory within the innate immune system. The occurrence of this immunomemory mechanism protects the individual against whether unrelated or homologous secondary infections. This process is known as trained immunity (TI) [135, 161]. The term of TI was firstly described in 2011 [135, 162]. A wide range of compounds including endogenous molecules (e.g., fumarate, uric acid, oxidized low-density lipoprotein ((oxLDL) (the oxLDL directly stimulates the glycoproteins of TLR4, by which the expression of IL-1*ß* increases), IL-1*ß*, IFN*γ* (CD8^+^ cell derived)) [135, 163–169], and exogenous microbial structures such as Bacillus Calmette-Guérin (BCG) (tuberculosis (TB)) vaccine, fungal *ß*-glucan (fungal cell wall constitution), and pathogenic protozoon of *Plasmodium falciparum* [135, 170–172]. Indeed, the TI promotes the biological activities of those innate immune cells which are involved in inflammatory comprising DCs, NK cells (and the related progenitor cells together with their hematopoietic stem cells) and monocytes (the circulating ones). Although the occurred TI adaptation covers a long-term protection between three and 12 months, it is reversible [135, 173–176]. As the reported results show, the triggered TI responses caused by the exogenous microbial inducers are considerably stronger than those occur by the endogenous stimuli [135, 177]. Interestingly, the TI is associated with promotion of anticancer immunity. In this regard, the BCG by the help of IT mechanism is used in immunotherapy for different types of cancers. The intravesicular application of BCG is the gold standard methodology for treating nonmuscular invasive bladder cancer (NMIBC). The application of BCG is also suggested as effective procedure for decreasing the risk of lymphoma and leukemia and treatment of malignant melanoma [178–182]. Although the IL-1 family members, in particular the IL-1*ß*, are identified as significant inducers of TI, there is a vital need for further studies to find out a logic relationship between the use of IL-1 family members and IT related treating procedures in association with treatment of inflammatory diseases [135, 183].

### 3.3. IL-1 (IL-1*α*/IL-1*ß*)

Both IL-1*α* (IL-1F1) and IL-1*ß* (IL-1F2) cytokines contribute to inflammatory responses, when they bind to IL-1R1. The *IL-1* gene (including *IL-1α* and *IL-1ß* genes) maps to the human chromosome 2q14. In contrast, inflammatory responses are blocked when they bind to IL-1Ra (IL-1F3). The *IL-1Ra* gene also maps to the human chromosome 2q14.2. IL-1*α* and IL-1*ß* cytokines are expressed by two different genes and share a low degree of sequence homology. The IL-1*α* precursor or pro-IL-1*α* is the biological active form of this cytokine, which acts as an alarmin. In this regard, the alarmin IL-1*α* is capable to induce the sterile inflammation through the activation of a cascade of inflammatory-related chemokines and cytokines. Indeed, the IL-1*α* is mostly a membrane-bound cytokine and that is why it is known as local cytokine. This is a specific property of IL-1*α*, which is an integral membrane protein (in particular in M*Φ*s). However, the nuclear IL-1*α* (nucleus located IL-1*α*) is associated with the transcription of proinflammatory molecules. Moreover, the pro-IL-1*α* may undergo the cleavage process via inflammatory proteases, e.g., the calcium-(Ca^2+^-) dependent protease of calpain, for further proinflammatory functions [2, 43, 68, 126, 128, 129, 135, 140, 184–187]. Oppositely once expressed, pro-IL-1*ß* is not biologically active.

The pro-IL-1*ß* is activated when the caspase-1 (a cysteine protease) cleaves it within the cellular cytoplasm or specific lysosomes. The caspase-1-dependent activation of pro-IL-1*ß* is induced through some inflammasome proteins, e.g., NOD-like receptor protein-1 (NLRP-1), NLRP-3, NLRP-4, NLRP-6, and NLRP-12. Indeed, the inflammasome is identified as a platform for IL-1*ß*, in which the IL-1*ß* gets matured and secreted from the related cells. Previous studies reveal that NLRP-3 inflammasome proteins are considerably contributed to IL-1*ß* processing. In parallel with the caspase-1-dependent activation of pro-IL-1*ß* (a cysteine protease), there is the caspase-1-independent activation of pro-IL-1*ß*, which belongs to serine proteases secreted by neutrophils [126, 128, 134, 140, 188–190]. In accordance with previous studies, expression of the IL-1*ß* is regulated by several regulatory regions scattered within thousands base pairs (bps) situated upstream from the starting site of transcription. Transcription of the inactive form of pro-IL-1*ß* is directly associated with binding of pattern recognition receptors (PRR), including TLR glycoproteins. The pro-IL-1*ß* is secreted in the presence of MAMPs, DAMPs, IL-1*α*, IL-1*ß*, IL-18, and TNF-*α*. IL-1ß has a pivotal role in the innate immune system through its effect on immune cells, including dendritic cells (DCs), M*Φ*s, and monocytes. It is presumed that IL-1*ß* is one of the main proinflammatory regulators in association with systemic immunoinflammatory responses. Any dysregulation in the active form of IL-1*ß* may result in a wide range of rare autoinflammatory diseases, e.g., Type 2 diabetes [126, 128, 134, 140, 168, 169, 191, 192]. Some other autoinflammatory diseases in which the secretion of IL-1*ß* gets considerably higher are as follows: chronic infantile neurologic, cutaneous and arthritis (CINCA) syndrome, familial cold autoinflammatory syndrome (FCAS), familial Mediterranean fever (FMF), Muckle-Wells syndrome (MWS), and neonatal-onset multisystem inflammatory disease (NOMID) [190, 193–199].

In accordance with previous studies, the IL-1*α*, IL-1*ß*, and IL-1Ra cytokine expressions increase in synovia of patients with rheumatoid arthritis (RA). Moreover, the level of IL-1Ra increases in the patient's serum with RA. Moreover, IL-1 is involved in development of cardiac remodeling and atherosclerosis [200–203]. The reported results reveal that the local activity of IL-1*ß* in the kidneys' podocytes of patients with SLE is associated with the downregulation of transcriptional process of the adhesion protein of nephrin. Nephrin supports the glomerular filter. Thus, the local activity of IL-1*ß* may lead to proteinuria and development of damages in the kidneys [203–206]. The IL-1*ß* cytokine is contributed to the inflammatory skin disease of psoriasis. Indeed, the expression of IL-1*ß* is promoted by the immune and nonimmune cells of DCs, M*Φ*s, and keratinocytes in appeared skin lesions in psoriasis [207].

### 3.4. IL-18

The *IL-18* gene maps to the human chromosome 11q22.2-11q22.3. The pro-IL-18 recalls pro-IL-1*ß*, being biologically inactive and becomes active via inflammasome's caspase-1. The well-known neutralizer of active IL-18I is L-18BP. The IL-18 (IL-1F4) is identified as strong inducer of the expression of IFN-*γ* by the immune cells of T_h1_, NK cells, and ILC1s in the presence of other ILs [2, 8, 43, 68, 128, 129, 187]. Indeed, productions of IFN-*γ* and other cytotoxic mechanisms are effective weapons for NK cells to remove the malignant cells [16, 208]. As different reports show, some cytokines comprising IL-10 and TGF-*ß* significantly reduce the secretion of activating NK cell receptors which is the outcome of tumor-mediated suppression [16, 209]. While the cytokines of IFN-*α* (alone), IL-2 (alone), IL-12 (alone), or combination of IL-12 and IL-18 support the promotion of cytotoxic activities of NK cells in patients with melanoma (in both stages of IIB and IIC). However, the single molecule of IL-18 has no influential effect on cytotoxic activities of NK cells [16].

IL-1*ß*, IL-18 may be processed by proteinase-3 in the extracellular space and has a specific decoy receptor. The IL-18-IL-18R*α* complex triggers the related signaling pathway together with MyD88 adaptor protein (myddosome), which may lead to the expression of NF-*κ*B [48, 128, 129, 210]. As previous reports indicate, IL-18 induces IFN-*γ* in the presence of IL-12, IL-15, and IL-21 within ILC1s. Moreover, the IL-18 molecules keep T_h1_- and cytotoxic T cells (CTCs) active. As IL-18 gets synergized together with IL-12 and T cell receptor (TCR), they trigger IFN-*γ* induction. This feature reveals the involvement of IL-18 in human protection against microbial agents and tumorigenesis, as well as its contribution to autoimmune interactions and damaging activities in different tissues [128, 211–214]. Due to this knowledge, IL-18 as a member of T_h1_ cytokines predominantly activates the fatal activities of immune cells of lymphocytes, M*Φ*s, and polymorphonuclear leukocytes (PMNs) against pathogenic viruses and bacteria [11, 160].

The IL-18 is determined as a proinflammatory immunomodulator. DCs, fibroblasts, M*Φ*s, NK cells, and functional B and T lymphocytes are the major expressive sources of IL-18 [8, 215]. The presence of soluble IL-18 (sIL-18) supports the production and secretion of CCR7 molecules upon the tumor-associated NK cells bearing CD56^dim^ molecules. The sIL-18 increases the movement of these NK cells into the SLOs and tertiary lymphoid structures (TLSs) [8, 216, 217]. The pro-IL-18, resembling IL-1*α* and IL-33, is perpetually secreted by different cells of the gastrointestinal tract. In this regard, keratinocytes, epithelial and endothelial cells are the main sources of IL-18 within the alimentary system. However, DCs and M*Φ*s are known as the frontline cells that produce active molecules of IL-18 [134].

Like IL-1*α*, IL-18 is bound to the cell membrane of monocytes and its pro-IL-18 tertiary structure has close similarity to IL-37. It seems that, this similarity is correlated with the flanking intron-exon regions of the genes encoding both ILs -18 and -37. These similarities explain why the IL-18R*α* accepts the IL-37 protein.

The levels of IL-18 in synovial fluid and sera of RA patients show increased concentrations. As the previous studies show, the IL-18 cytokine has high biologic activity in synovial fluid and sera of patients with RA. The IL-18 is also associated with autoimmune and chronic inflammatory diseases, such as acute kidney injury, cardiovascular disease, Crohn's disease, psoriasis, respiratory diseases (e.g., asthma and chronic obstructive pulmonary disease), sepsis, SLE, and type I diabetes [68, 126, 129, 134, 157, 203, 218–220].

As the results show, the IL-18 and IL-18BP are highly expressed in the sera of patients with SLE. Furthermore, in parallel with the highly expressed IL-18BP, the level of overall free IL-18 is increased in the patients, too. In accordance with clinical findings, the overexpression of IL-18 leads directly to the active nephritis in patients with SLE. Therefore, the IL-18 contributes to SLE pathogenesis and inflammation mediation during the active phase of the disease [77, 203, 221]. It is estimated that the IL-18 contributes to differentiation and proliferation of the local T cells and migration of cells into the kidneys. The overexpression of IL-18 is detected in both tubular epithelial cells and the infiltrated mononuclear cells in glomeruli. Interestingly, the IL-18 cytokines employ those plasmacytoid dendritic cells that are able to secrete IL-18R. The IL-18 as multifunctional cytokine regulates the T cell subsets in patients with lupus nephritis [203, 222–224].

As reported results from previous studies show, IL-18 and IL-1*ß* are detected in high level of brain tissue, circulation and cerebrospinal fluid (CSF) of patients with multiple sclerosis (MS). MS is known as CNS-associated autoimmune disease in which neuroinflammatory and neurodegenerative features occur and result in serious neurological damages in individuals. High levels of mRNA molecules belonging to IL-18 and caspase-1 in peripheral blood mononuclear cells (PBMCs) are detectable in patients with MS. Moreover, the production of caspase-1 highly increases in both plaques of acute and chronic diseases in patients with MS. Due to this fact, the levels of caspase-1 enzymes in the sera of individuals can be identified as effective diagnostic method for MS, because the caspase-1 enzymes can be recognized as biomarkers [203, 225–232].

### 3.5. IL-33

The *IL-33* gene maps to the human chromosome 9p24.1. The pro-IL-33 is biologically active and any enzymatic processing on it can occur in the extracellular milieu. The cathepsins, proteases, and elastase secreted by both neutrophils and mast cells process pro-IL-33 extracellularly, thereby considerably promoting IL-33 (IL-1F11) activity. IL-33 is situated in the nuclear zone of the cells in homeostasis condition. When IL-33 is secreted by damaged cells and no enzymatic processing takes place, it is still biologically active but it is known as an alarmin or DAMP [2, 43, 68, 126, 129, 134, 187, 233, 234]. Although IL-33 is considered as an anti-inflammatory cytokine, it carries the same coreceptor (IL-1R3) as IL-1*α* and IL-1*ß* do. The bond of IL-33 to its receptor “IL-1R4 (ST2)” generates a spatial change in its configuration, as both IL-1*α* and IL-*ß* do. However, the complex of IL-33-IL-1R4 (ST2) does not lead to a signaling pathway. The obtained changes in spatial configuration of IL-33-IL-1R4 (ST2) may lead to a further binding to make a new heterotrimeric complex, made of IL-33-IL-1R4 (ST2)-IL-1R3, which triggers the signaling pathway in which the MyD88 adaptor and IRAKs are recruited to express the NF-*κ*B. The sST2 is identified as decoy receptor and negative regulatory protein which is involved in IL-33 signaling pathway [68, 134, 235, 236].

The IL-33 molecule contributes to some effective defensive structures including the gut, mucosal linings, and lung. The intracellular pro-form of IL-33 regulates the transcriptional processes and the extracellular cleaved and processed form of IL-33 is identified as a soluble cytokine. In addition, the intracellular cytokine of IL-33 is contributed to epigenetic regulation and downregulation of NF-*κ*B, IL-18, and IL-6 expression and/or activity [190, 237].

As an axial cytokine, IL-33 mediates and regulates the activities of ILC2, T_h2_, regulatory T (T_reg_) cells, M*Φ*s, NK cells, and DCs and degranulation of basophils, eosinophils, and mast cells, belonging to type 2 innate and adaptive immune systems and inflammatory immune responses. The IL-33 has pivotal role in association with allergic inflammatory responses and it is involved in protective mechanisms against asthma, *Toxoplasma gondii* (encephalitis) and helminthic infections. These mechanisms are supported by the induction of IL-13 within the ILCs. In contrast, a suppressive role of IL-1*ß* against helminth infections by inhibiting IL-25 and IL-33 cytokines secretion by preventing the clearance activities against helminth pathogens and leading to chronic infections was identified [236, 238–243]. As clinical findings show, the IL-33 is considerably down-expressed, while the sIL-1R4 which is the inhibitor of IL-33 is overexpressed in patients with SLE [203, 244]. According to reported results obtained from clinical findings, the IL-33 contributes to acute gut inflammation in patients with inflammatory bowel disease (IBD); however, it has protective and reparative function in the chronic phase of the IBD [203, 245].

As the results show, although the level of IL-33 together with IL-1*ß* and IL-18 in the CSF, CNS and sera of patients with MS highly increase, the role of IL-33 in the pathogenesis mechanism of MS is not clear [203, 246, 247].

### 3.6. IL-36

The optimal activity of IL-36 cytokines is achieved via enzymatic processing performed by proteolytic activity of the caspase-1, independently from the presence of protease enzymes expressed by neutrophils [248]. The IL-36 cytokine belongs to the IL-36 subfamily within the IL-1 superfamily, which is composed by three agonists (comprising IL-36*α* (IL-1F6), IL-36*ß* (IL-1F8), and IL-36*γ* (IL-1F9)) and one antagonist (IL-36Ra (IL-1F5)). The specific receptor for these members is the heterodimeric complex of IL-36R (IL-Rrp2)-IL-1RAcP [139, 146, 249, 250]. The *IL-36A, IL-36B, IL-36G*, and *IL-36Ra* genes map to the human chromosomes of 2q12-2q14.1, 2q14, 2q12-2q21, and 2q14, respectively [2, 43, 129, 187, 251, 252].

IL-36 cytokines are secreted by immune and nonimmune cells including fibroblasts, epithelial cells, neural cells, keratinocytes, DCs, and M*Φ*s. Those cells which express IL-36 cytokines and belong to innate and adaptive immune systems trigger different cytokines, such as proinflammatory cytokines which result in proliferation of T_h1_ and polarization of T_h17_ [248, 250, 253, 254]. Moreover, IL-36 contributes to skin immunopathological features, e.g., psoriasis and fungal respiratory immunopathological conditions caused by *Aspergillus fumigatus* and kidney diseases. In accordance with reported results, these diseases are resulted from the excessive expression or activity of the IL-36 [129, 190, 255, 256]. As the reported results show, the expression of IL-36 cytokine in the synovia of patients with RA significantly increases [203, 257]. Furthermore, the IL-36*α* and IL-36*γ* isoforms are increased in the sera of patients with active SLE [203].

According to clinical findings, the IL-36 is highly active in psoriasis disease. Although the IL-36*α* and IL-36*ß* isoforms together with IL-38 are produced in normal condition of healthy skin, the production of IL-36*α* and IL-36Ra together with IL-38 promotes during psoriasis within the psoriatic lesions. Simultaneously, the presence of IL-36*γ* has been detected within the psoriatic lesions. The IL-36 molecules are secreted through different cells of Langerhans, keratinocytes, and M*Φ*s and these cytokines simultaneously are able to be amplified by themselves autocrine loop. The immune- and resident skin cells are both identified as target cells for IL-36 cytokines. Induction of IL-36 cytokines leads to secretion of high levels of inflammatory mediators via epidermal keratinocytes, activation of endothelial cells, and triggering neoangiogenesis. The IL-36 involves in DC maturation, T_h1_ cell induction, and appearance of M1 phenotype M*Φ*s [203, 258, 259].

### 3.7. IL-37

The *IL-37* gene maps to the human chromosome 2q12-2q14.1. Among five isoforms identified for IL-37 (a-e), the IL-37b or isoform 1 is known as the most complete [2, 43, 129, 187, 260]. The pro-IL-37, like IL-1*α* and IL-33, is biologically active; however, this cytokine can be enzymatically processed, too. The complex of IL-37-IL-18R*α* (receptor)-IL-1R8 (coreceptor) represents the anti-inflammatory characteristic of IL-37. The presence of an instability sequence in the *IL-37* gene results in a significant reduction of IL-37 (IL-1F7) molecules in DCs and blood monocytes in human being. Therefore, the inducing agents comprising TGF-*ß*, TLR agonists and ligands, and IL-1*ß* are needed to promote the expression of IL-37 by blood monocytes in humans. Moreover, the IL-37 is secreted by M*Φ*s in different tissues, plasma cells, tonsillar B lymphocytes, epithelial cells regarding the intestine, kidney, and skin [2, 68, 129, 134].

Three cytokines, belonging to the IL-1 superfamily including IL-37, Il-33, and IL-1*α*, are able to bind to nuclear DNA and translocate to the cell's nucleus. Thus, these cytokines are involved in transcriptional processes. Due to this knowledge, the IL-37, Il-33, and IL-1*α* cytokines are called as dual functional cytokines. The IL-37 is able to decrease the expression of proinflammatory cytokines which is induced by LPS in mice [68, 134, 261, 262]. As aforementioned, the long protein of pro-IL-37 is intracellularly functional and contributes to regulate the transcriptional activities which results in promotion of anti-inflammatory activities. Interestingly, the processed IL-37 (cleaved by enzymes) is detected as soluble cytokine resembling its proform act as anti-inflammatory cytokine [203, 263, 264].

It is shown that the level of IL-37 in the plasma and peripheral lymphocytes of patients with RA increases [203, 265]. In addition, the level of IL-37 is high in sera of patients with SLE and directly is related to the disease condition and activity (in particular at the renal level) [203, 266, 267].

The IL-37 cytokine is involved in MS disease, too. As reported results reveal, the level of IL-37 increases in patients with relapsing-remitting multiple sclerosis (RRMS) and neuromyelitis optica (NMO). In another word, the level of IL-37 cytokine in serum of patients with MS determines the severity and condition of the MS disease [203, 268, 269].

### 3.8. IL-38

Indeed, IL-38 (IL-1F10) is an in silico identified cytokine, which resulted from bioinformatic investigations of the human genome. The *IL-38* gene maps to the human chromosome 2q14, adjacent to the *IL-1Ra* and *IL-36Ra* genes. The spatial configuration of IL-38 resembles the IL-1Ra's, although IL-38 has less than 50% homology with IL-1Ra (41%) and IL-36Ra (43%). IL-38 is able to bind to IL-1R type I, IL-1R accessory protein-Fc, IL-18 receptor *α* chain-Fc, and IL-1Rrp2-Fc (IL-36R). It seems that IL-38 regulates the production of T_h17_ cells, the mediator of immune responses due to their potentiality to produce proinflammatory cytokines, including IL-17A, IL-17F, and IL-22. T_h17_ participates in pathogenesis of variety of inflammatory and autoimmune diseases [2, 43, 68, 129, 187, 270–272].

By suppressing generation of proinflammatory cytokines followed by reduction in Th17 maturation, IL-38 may contribute to inflammatory diseases such as rheumatic autoimmune diseases (i.e., SLE, RA, and psoriasis), Primary Sjögren's Syndrome (PSS), Chronic Obstructive Pulmonary Disease (COPD), Oxygen-Induced Retinopathy (OIR), inflammatory bowel diseases (IBD), and autoimmune thyroid diseases [203, 257, 273].

### 3.9. *IL-1* Gene Cluster and Reported Polymorphisms

**Figure 2:**
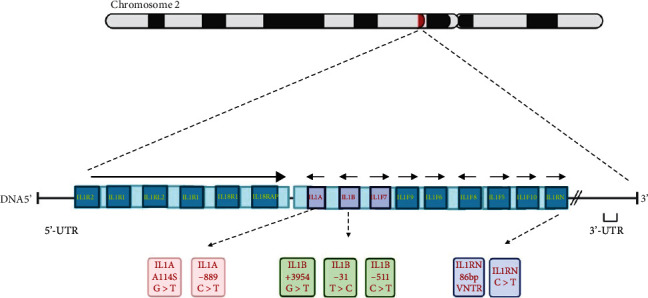
Map of *IL-1* gene clusters on chromosome 2. Putative functional SNPs are annotated for IL1A, IL1B, and IL1RN (https://Biorender.com, modified) [124, 274].

The IL-1 family of cytokines consists of a cluster of classical cytokine agonists (IL-1*α*, IL-1*ß*, IL-18, IL-33, IL-36*α*, IL-36*ß*, and IL-36*γ*), the receptor antagonist (IL-Ra, IL-36Ra, and Il-38), and an anti-inflammatory cytokine (IL-37). The majority of the *IL-1* gene family is located on chromosome 2 in two distinct clusters (Figure 2) and is reported to contain about 1,500 SNPs [274].

### 3.10. IL-1*α* SNPs

The *IL*-*1α* gene consists of seven exons and six introns and is expressed as a 31-kDa protein. About 148 SNPs have been reported for this gene, but only a few are studied [275]. One of the most studied polymorphisms of the *IL-1α* gene (rs17561) is located at position +4,845 with respect to the gene locus. It causes the missense substitution of guanosine (G) to thymine (T), resulting in the change of Alanine to Serine [276]. So, the SNP in *IL1* gene including -889C/T in rs1800587 is related to Alzheimer's disease [276–278] and Primary Open Angle Glaucoma (POAG) [279]; however, the correlation between IL1A SNP and POAG has not been detected [280] (Table 1).

Another important SNP (rs1800587) is located in the promoter region at a position -899, resulting in the substitution of cytosine (C) to thymine (T) (*C>T*). It was reported that T allele for IL-1*α* SNP (rs1800587) is associated with the higher expression of IL-1*α* in transfected pancreatic cell [276]. Additionally, it was reported that TT genotype had a higher mRNA level in comparison to CC or Ct genotype, which resulted in a significantly higher IL-1*α* serum levels (TT = 36pg/ml vs. CC = 6.8pg/ml) [274, 276] (Table 1).

For instance, evidence from several meta-analyses revealed that IL-1*α* SNP, rs1800587 (-889C/T) is significantly associated with the risk of chronic periodontitis (CP) therefore plays an important role in CP pathogenesis [281, 282]. Periodontitis, traditionally classified as either aggressive periodontitis (AgP) or CP, is an infection-driven inflammatory disease in tooth-supporting tissues initiated by specific species of microorganisms [283–285]. Analyses of IL-1*α* C-889T (rs1800587) and IL-1*α* G+4845T (rs17561) genotype distributions among healthy obese patients revealed that both IL-1*α* polymorphisms were associated with an increase in body mass index (BMI) [286]. Additionally, IL-1*α* C-889T (rs1800587) has been shown to be critical for the development of obesity by affecting the transcriptional activity of IL-1*α* in preadipocyte 3T3-L1 cell line model [286] (Table 1).

### 3.11. IL-1*β* SNPs

*IL-1β* gene is about 7.5 kb long, contains seven exons, and it is regulated by 5' and 3'–UTR regions [287, 288]. The *IL*-*1β* gene is the second gene displaying several polymorphisms in its sequence, with almost 144 SNPs reported [275]. Two of the most common SNPs of the *IL-1β* gene that have been extensively reported are within the coding region, at position +3,954 affecting exon 5 (*rs1143634; C>T)*, and two within the promoter region, at position -31 *(rs1143627; T>C)* and -511 *(rs16944; C>T)* [287, 289] with respect to the +1 position of the gene. The T allele of IL-1*β*  (*rs1143634; C>T)* SNP is rarer than the С allele and has been reported to result in elevated serum IL-1*β* levels [290]. The SNP -31 *(rs1143627; T>C)* within the promoter was demonstrated to cause disruption of the TATA box upstream of the transcription start site, thereby leading to changes in the expression of the gene [287] (Table 1).

The T allele of IL-1*β*  (rs1143634; C>T) SNP rarer than the С allele has been reported, resulting in an elevated serum IL-1*β* levels and possible association with higher risk of lung cancer especially among smokers [291]. This polymorphism has also been found to be associated with an increased risk of CP [292–294]. High levels of IL-1*β* have been reported in the gingival crevicular fluid (GCF) of individuals with IL-1*β* rs1143634 polymorphism that might be a genetic risk factor for periodontitis [295]. However, some studies do not support such an association, especially in aggressive periodontitis [296, 297]. A recent study reported that compared to wild-type CC genotype, this polymorphism with CT genotype (*P* = 0.02) has been linked in development of Autism Spectrum Disorder (ASD) in Turkish (Turkey) children [298]. Although no significant difference in the frequency of *IL1β* +3954 C/T (rs1143634) gene polymorphism was found between SLE patients and the control group, Behiry et al. reported that serum levels of IL1*β* in SLE cases increased gradually in rs1143634 TT, CT, and CC genotypes with CC genotype at the highest level (median 46.6 pg/mL; range 43.1–404.4 pg/mL) [299] (Table 1).

Analysis of functional polymorphisms in the *IL1β* gene showed that the -511 (rs16944; C→T) and -31 (rs1143627; T→C) polymorphisms affect IL-1*β* production. The promoter SNP -31 (rs1143627; T→C) is demonstrated to cause disruption of the TATA box upstream of the transcription start, leading to the change in expression of the gene [287]. Moreover, T allele of this polymorphism has been associated with higher nuclear protein binding than the -31C allele in lipopolysaccharide-activated monocytes [300]. An in vitro study on A549 lung cancer cells confirmed that -31 T allele had a significantly higher promoter activity than the −31 C allele [301]. However, Fu et al. reported that carriers of IL-1B rs1143627 genotypes AA had a higher risk of coronary artery lesions (CALs) in Kawasaki disease (KD) than respect to those carrying either GG or AG genotypes that might significantly impact the risk of CAL formation in children younger than 12 months [302] (Table 1).

The promoter SNP locus (rs16944; C→T) is located in a regulatory motif, facilitating interactions with transcriptional factors, leading an increase activity of the *IL-1β* gene promoters. Moreover, this polymorphism controls IL-1*β* transcription of TT and CT genotypes, which have been involved in IL-1*β* overproduction in inflammation-related conditions, such as bladder, colon, breast, and lung cancer [303]. A previous study revealed that the C allele of rs16944 (*P* = 0.022) and the T allele of rs1143627 (*P* = 0.025) were associated with a significantly increased risk of keratoconus in Korean patients [304]. Keratoconus is an asymmetric and noninflammatory coronal disorder that leads to corneal thinning, protrusion, and irregular astigmatism [305, 306]. IL-1*β* polymorphism (rs16944 T/C) is also associated with cutaneous leishmaniasis caused by *Leishmania guyanensis* with C/C as common genotype among the patients (*P* = 0.004) and C allele associated with susceptibility to *L. guyanensis* infection (*P* = 0.003) [307] (Table 1).

### 3.12. IL-1RN SNPs

The classical *IL-1RN* gene has four exons and, currently, about 140 SNPs have been recorded for this gene [275]. The earliest reports and the most common polymorphisms of this gene were described as a penta-allelic 86 bp variable number tandem repeat (VNTR), located within intron 2 of the *IL10Ra* gene or within intron 3 of the extended gene containing the additional 5′ exon encoding IL1-Ra1 [308, 309]. For this VNTR, IL1RN∗1 contains four repeats of the 86 bp in tandem, while IL1RN∗2 two repeats, and IL1RN∗3 five repeats [310]. It was reported that IL1RN∗1 homozygotes release more IL-1*β* in serum than subjects who carry at least one IL1RN∗2 allele, which is consistent with a functional genotype effect in that IL1RN∗2 is associated with higher IL-1Ra release [311]. The other common reported polymorphism in *IL-1RN* gene is located at position *+2018 (rs419598)* in the exon 2 of the *IL-1RN* gene, that might be associated with a reduced susceptibility to generalized aggressive periodontitis (GAgP) and generalized chronic periodontitis (GCP) in populations of European descent [312]. Nevertheless, an overview of previous studies indicates no association between this polymorphism and periodontitis risk in populations of European descent [313, 314]. Hence, there is inconsistent evidence on IL-1 genetic risk factors for periodontitis (Table 1).

In another study which was performed by Abbasian et al., the correlation between IL-1RN VNTR and colorectal and gastric cancers and the correlation between rs419598 polymorphisms and colorectal and gastric cancers (among Iranian population) were investigated. The results revealed that the risk of colorectal and gastric cancers among those individuals who carried IL-1RN∗ 2 allele was increased. In addition, the risk of gastric cancer among those individuals who carried homozygous ILRN ∗2/∗2 genotype was increased. However, no correlation between the alleles of rs419598 and the colorectal and gastric cancers was detected [315]. Recently, Ciurla et al. in their study have shown that the presence of purinoreceptor (*P2RX7*) (rs208294) and *IL1RN* (rs419598) SNPs are known as predisposing factors to external apical root resorption (EARR) in individuals. This result indicates that the EARR may have genetic predisposing factors rather than environmental factors [316].

In another study which was achieved by Attur et al. the results show that the presence of *IL1RN* TTG haplotype in individual is correlated with more severe radiographic osteoarthritis (rOA) compare with the individuals who do not carry *IL1RN* TTG haplotype with the same BMI, age, and sex. The risk of incidence for rOA increases with 4.1-fold among those who carry *IL1RN* TTG haplotype [317]. Furthermore, the IL-1Ra levels in *IL1RN* TTG haplotype carrier's plasma are lower and simultaneously their chondrocytes secrete the IL-1Ra in low level. In toto, in patients with rOA who carry *IL1RN* TTG haplotype show higher level of disease activity (DAS28) and plasma inflammatory markers, e.g., high-sensitivity C-reactive protein (hsCRP) and interleukin 6 (IL-6). Both haplotypes of CTA and TTG are resulted from the occurrence of three SNPs of rs419598, rs315952, and rs9005 in the *IL1RN* gene [317] (Table 1).

### 3.13. IL-18 SNPs

IL-18 was originally termed as IFN-*γ*-inducing factor and classified as an extended member of the IL-1 superfamily [274, 318] (Table 1). The *IL-18* gene is located in the long arm of chromosome 12 (12q22). Among the various SNPs that have been reported for the *IL-18* gene, most of them have been detected in the promoter regions (-5848T>C,1297 T/C, -667G/T, -656T/G, -607A/C, -148G/C; -137G/C), upstream of exon 1 and 2 of the gene. The others were reported within the gene itself (+8925C/G, +13925A/C) [318–321]. Among these, three IL-18 SNPs -5848T>C, -607A/C, and -137G>C have been associated significantly with the variation in gene expression and hence with lower level of IL-18 [274, 319, 322].

Kou et al. achieved an investigation. They worked on the correlation between the effect of IL-18 and the development of osteoporosis. In this regard, 8 SNPs of IL-18 including rs6760105, rs6748621, rs7577696, rs2250417, rs212713, rs2300702, rs2268797, and rs212745, the first four SNPs of IL-18 had weak effects on IL-18, while the second four SNPs had strong effects on IL-18. The results revealed that the risk of the development of osteoporosis may increase through the reduction of IL-18 level. However, more investigations are needed [323].

### 3.14. IL-33 SNPs

IL-33 is a relatively new cytokine of the IL-1 family, which serves as the ligand of the IL-1RL1 (ST2) receptor [324] (Table 1). The *IL-33* gene is located in chromosome 9p24.1, containing 11 exons which encode a 30 kDa IL-33 protein [325]. IL-33 is a critical mediator of allergic immune responses and has been demonstrated to play key role in multiple allergic and inflammatory diseases [326–328]. A total of eight polymorphisms have been reported for the *IL-33* gene till date, and all have been implicated in asthma susceptibility (Table 2). Several of the IL-33 SNPs lie in close proximity to each other and hence result in the LD [324].

## 4. Conclusion

Interleukins (ILs) constitute an important subgroup of cytokines and biological molecules of critical importance and with wide-ranging functions in both health and disease. The IL-1 superfamily is characterized by specific structural motifs, and among its eleven members, it has both agonists and antagonists within its ranks, bearing critical functions in maintaining the functions of the innate and adaptive immune system. With the advent of modern technologies in molecular biology and genetics, researchers have the ability to provide more detailed insights into the biological variability of ILs than previously thought possible. The occurrence of single nucleotide polymorphisms (SNPs)—which are considered as the most common genetic variation among individuals—in IL-encoding genes may have considerable effect on the function of ILs, thus, leading to pronounced pathophysiological consequences for humans. In our review, we have summarized the most important biological-immunological functions of the IL-1 superfamily of cytokines and provided insights on the relevance of SNPs affecting these ILs, leading to increased susceptibility to some infectious agents, higher risk for noncommunicable illnesses, and the development of immunological pathologies. As the genomic platforms used to study the human genome and SNPs become more prevalent and high-throughput, we may expect a surge of information related to the presence of SNPs in the IL-1-superfamily genes and the presentation of diseases. If relevant stakeholders in clinical practice could effectively uptake this information, this could be used as a basis for pharmacogenomics, disease forecasting and improving patient care globally.

## Figures and Tables

**Table 1:** The most important SNPs detected in the IL-1 family members.

ILs	SNPs	Chromosome Location	Allele	SNP Class	Type of Disease	References
IL-1*α*	rs17561	2:112779646 (GRCh38), 2:113537223 (GRCh37)	C>A	missense	Increase in BMI among the healthy obese women	[275, 276, 286], (https://www.ncbi.nlm.nih.gov/snp/?term=IL-%CE%B1+rs17561)
	rs1800587	2:112785383 (GRCh38) 2:113542960 (GRCh37)	G>A / G>C / G>T	Single Nucleotide Variation (SNV)	Alzheimer's disease, chronic periodontitis, increase in BMI among the healthy obese women, and higher expression of IL-1*α* in transfected pancreatic cell	[276–280, 282], (https://www.ncbi.nlm.nih.gov/snp/?term=rs1800587)
IL-1*ß*	rs1143634	2:112832813 (GRCh38),2:113590390 (GRCh37)	G>A	SNV	The increase of serum IL-1*β* level, possible association with higher risk of lung cancer especially among smokers, increased risk of CP, a genetic risk factor for periodontitis, some linkages between this SNP and the development of ASD among Turkish (Turkey) children are recognized, and increase in serum levels of IL1*β* in SLE patients	[291–295, 298, 299](https://www.ncbi.nlm.nih.gov/snp/?term=rs1143634)
	rs1143627	2:112836810 (GRCh38)2:113594387 (GRCh37)	G>A	SNV	carriers of IL-1B rs1143627 genotypes AA had a higher risk of CALs in KD	[302](https://www.ncbi.nlm.nih.gov/snp/?term=rs1143627)
	rs16944	2:112837290 (GRCh38) 2:113594867 (GRCh37)	A>G	SNV	This SNP is involved in IL-1*β* overproduction in inflammation-related conditions, such as bladder, colon, breast, and lung cancer, increasing the risk of keratoconus in Korean patients, associated with cutaneous leishmaniasis caused by *Leishmania guyanensis*	[303, 304, 307](https://www.ncbi.nlm.nih.gov/snp/?term=rs16944)
IL-1RN	rs419598	2:113129630 (GRCh38)2:113887207 (GRCh37)	T>C	SNV	might be associated with a reduced susceptibility to GAgP and GCP in populations of European descent, increasing more severe rOA,	[312, 317](https://www.ncbi.nlm.nih.gov/snp/?term=rs419598)
	rs315952	2:113132727 (GRCh38)2:113890304 (GRCh37)	T>A,C	SNV	increasing more severe rOA,	[317](https://www.ncbi.nlm.nih.gov/snp/?term=rs315952)
	rs9005	2:113133835 (GRCh38)2:113891412 (GRCh37)	G>A	SNV	increasing more severe rOA,	[317](https://www.ncbi.nlm.nih.gov/snp/?term=rs9005)
IL-18	More investigations are needed					
IL-33	More investigations are needed					

**Table 2:** Some important IL-33 SNPs associated with asthma.

SNPs	Chromosome Location	Allele	SNP Class
rs1342326	6.190.076	C^z^T (with rs928413G)	5′
rs2381416	6.193.455	C	5′
rs3939286	6.210.099	A^z^A	5′
rs928413	6.213.387	G (with rs1342326T)	5′
rs2066362	6.219.176	T^z^	Intron
rs16924159	6.229.417	A^y^	Intron
rs12551256	6.231.239	G^y^	Intron
rs7025417	6.240.084	C^z^	Intron
